# The Transcription Factor Ste12 Mediates the Regulatory Role of the Tmk1 MAP Kinase in Mycoparasitism and Vegetative Hyphal Fusion in the Filamentous Fungus *Trichoderma atroviride*

**DOI:** 10.1371/journal.pone.0111636

**Published:** 2014-10-30

**Authors:** Sabine Gruber, Susanne Zeilinger

**Affiliations:** Research Area Biotechnology and Microbiology, Institute of Chemical Engineering, Vienna University of Technology, Wien, Austria; Georg-August-University of Göttingen Institute of Microbiology & Genetics, Germany

## Abstract

Mycoparasitic species of the fungal genus *Trichoderma* are potent antagonists able to combat plant pathogenic fungi by direct parasitism. An essential step in this mycoparasitic fungus-fungus interaction is the detection of the fungal host followed by activation of molecular weapons in the mycoparasite by host-derived signals. The *Trichoderma atroviride* MAP kinase Tmk1, a homolog of yeast Fus3/Kss1, plays an essential role in regulating the mycoparasitic host attack, aerial hyphae formation and conidiation. However, the transcription factors acting downstream of Tmk1 are hitherto unknown. Here we analyzed the functions of the *T. atroviride* Ste12 transcription factor whose orthologue in yeast is targeted by the Fus3 and Kss1 MAP kinases. Deletion of the *ste12* gene in *T. atroviride* not only resulted in reduced mycoparasitic overgrowth and lysis of host fungi but also led to loss of hyphal avoidance in the colony periphery and a severe reduction in conidial anastomosis tube formation and vegetative hyphal fusion events. The transcription of several orthologues of *Neurospora crassa* hyphal fusion genes was reduced upon *ste12* deletion; however, the Δ*ste12* mutant showed enhanced expression of mycoparasitism-relevant chitinolytic and proteolytic enzymes and of the cell wall integrity MAP kinase Tmk2. Based on the comparative analyses of Δ*ste12* and Δ*tmk1* mutants, an essential role of the Ste12 transcriptional regulator in mediating outcomes of the Tmk1 MAPK pathway such as regulation of the mycoparasitic activity, hyphal fusion and carbon source-dependent vegetative growth is suggested. Aerial hyphae formation and conidiation, in contrast, were found to be independent of Ste12.

## Introduction

Mycoparasitic species of the fungal genus *Trichoderma* are potent biocontrol agents and promising substitutes for chemical fungicides as they attack and parasitize plant pathogens, such as *Rhizoctonia spp.*, *Phythium spp.*, *Botrytis cinerea* und *Fusarium spp.* [1]. Mycoparasitic responses are triggered by molecules released from the host fungus and through physical contact accomplished through surface located components (e.g. lectins) [2], [3]. As a consequence, *Trichoderma* inhibits or kills the host by parasitizing its hyphae thereby employing hydrolytic enzymes like chitinases, proteases, and glucanases which degrade the host's cell wall. Mycoparasitism further includes shaping of infection structures (coiling response) and the production of antimicrobial secondary metabolites [4]. In the past years, investigation of signaling pathways in the potent mycoparasites *Trichoderma atroviride* and *Trichoderma virens* showed essential roles of conserved signaling routes involving G protein-coupled receptors (GPCRs) and heterotrimeric G proteins, the cAMP pathway and mitogen-activated protein kinase (MAPK) cascades in regulating vegetative growth, conidiation, and mycoparasitism-associated processes (reviewed in [5], [6].

MAPK cascades are characterized by a three-tiered signaling module comprising a MAPK kinase kinase (MAPKKK), a MAPK kinase (MAPKK) and the MAPK which is hierarchically activated by dual phosphorylation of conserved threonine and tyrosine residues [7]. The proposed mechanism of MAPK signaling comprises the transduction of extracellular and intracellular signals, thereby often regulating transcription factors by MAPK-mediated phosphorylation. Fungal MAPKs are involved in regulating a wide range of processes including cell cycle, stress response and several essential developmental processes such as sporulation, mating, hyphal growth, and pathogenicity [8], [9]. In the yeast *Saccharomyces cerevisiae*, mating and filamentous growth are controlled by the Fus3 and Kss1 MAPKs, respectively [10]. Despite their distinct activation mechanisms and signaling output, both MAPKs target the homeodomain transcription factor Ste12, which acts as a central node in both mating and invasive growth and that is under complex regulation by several regulatory proteins and co-factors being tightly controlled by each MAPK. The Fusp/Kss1 MAPK cascade is highly conserved in filamentous fungi which, however, in most cases only posses a single Fus3/Kss1 orthologue [11]. Δ*mak-2* mutants of the model fungus *Neurospora crassa* showed reduced growth rate, derepressed conidiation, failed to develop protoperithecia, and lacked hyphal fusion – phenotypes which they share with Δ*pp-1* mutants missing the *ste12* homologue [12]. In the phytopathogenic fungus *Magnaporthe oryzae*, the Fus3/Kss1 homologous MAP kinase Pmk1 is essential for pathogenicity-related processes. Δ*pmk1* mutants failed to form appressoria and to grow invasively in plants but still recognized hydrophobic surfaces [13]. Studies from several phytopathogenic fungi, including appressorium- and non-appressorium-forming pathogens, necrotrophs and biotrophs, revealed a conserved role of the Pmk1 MAPK pathway for regulating plant infection with respective deletion mutants being affected in pathogenicity-related processes such as appressorium formation, penetration hyphae differentiation, root attachment and the production of plant cell wall-degrading enzymes (reviewed in [9,14]). Concordant with the model of Ste12 being targeted by the Fus3/Kss1 homologous Pmk1-type MAP kinase, *ste12*-deficient mutants of several phytopathognic fungi are either non-pathogenic or suffer from strongly attenuated virulence (reviewed in [11], [15]).

Similar to other fungal pathogens, the molecular processes involved in host attack in mycoparasitic fungi are tightly regulated by conserved signaling pathways. In both, *T. atroviride* as well as *T. virens*, the Pmk1 MAPK homologues Tmk1/TmkA (Tvk1) play crucial, albeit species-specific, roles in mycoparasitism [16]–[18]. *T. virens* Δ*tvk1*/Δ*tmkA* mutants showed secondary metabolite production similar to the wild-type and unaltered mycoparasitism of *R. solani*, while antagonism against *Sclerotium rolfsii* was reduced [16], [18]. In contrast, deletion of *tmk1* in *T. atroviride* resulted in mutants with reduced mycoparasitic activity against *R. solani* and a loss of mycoparasitism of *B. cinerea* although Δ*tmk1* mutants showed an increased production of antifungal metabolites such as peptaibols and 6-pentyl-α-pyrone [17].

Although recent comparative genomic analyses revealed structural conservation of Fus3/Kss1 MAPK cascade components in taxonomically and biologically diverse fungi [19], [20], the available studies showed remarkable functional differences between fungi with phytopathogenic and mycoparasitic lifestyles. While in plant pathogens such as *Fusarium oxysporum* and *Cochliobolus heterostrophus* the expression of extracellular plant-lysing enzymes is positively regulated by the Pmk1-type MAPK [21], [22], Tmk1 and Tvk1 repress the production of secreted mycoparasitism-relevant cell wall-lysing chitinases and proteases in the mycoparasites *T. atroviride* and *T. virens* [16], [17]. A further dissection of the Fus3/Kss1 MAPK cascade including detailed analyses of factors acting upstream and downstream of the core MAP kinase and discovery of the signals originating from the respective hosts will be necessary for a detailed understanding of this widely conserved signaling pathway in different fungi.

The objectives of this study were to confirm the presence of a functional Ste12 homolog in the mycoparasite *T. atroviride* and to characterize its function as an assumed central component of the mycoparasitism-relevant Tmk1 MAPK signaling pathway. To this end, we deleted the *ste12* gene and comparatively analyzed *T. atroviride* Δ*ste12* and Δ*tmk1* mutants regarding physiological and differentiation processes. Furthermore, the role of Ste12 in host sensing and mycoparasitism of *T. atroviride* was addressed. Our study provides the first functional characterization of a Ste12-like transcription factor in a fungus exhibiting a mycoparasitic lifestyle and unveils the Tmk1-Ste12 signaling pathway as key player not only in mycoparasitism but also hyphal avoidance, vegetative hyphal fusion and carbon source-dependent growth of *T. atroviride*.

## Materials and Methods

### Cultivation conditions

*T. atroviride* strain P1 (ATCC 74058; teleomorph *Hypocrea atroviridis*), was used in this study. The parental as well as the mutant strains Δ*ste12* and Δ*tmk1-12* [23] were cultivated at 28°C using a 12 hours light/dark cycle in either rich medium (potato dextrose agar, PDA, or potato dextrose broth, PDB) (BD Dicfo, Franklin Lakes, NJ), or minimal medium (MM, containing [g/l]: MgSO_4_·7H_2_O 1, KH_2_PO_4_ 10, (NH_4_)_2_SO_4_ 6, tri-sodium citrate 3, FeSO_4_·7H_2_O 0.005, ZnSO_4_·2H_2_O 0.0014, CoCl_2_·6H_2_O 0.002, MnSO_4_·6H_2_O 0.0017, glucose or glycerol 10). Cultivations in liquid medium were either performed in stationary cultures or shake flask cultures, depending on the respective experiment. For testing hyphal network formation, liquid stationary cultures were inoculated with an agar plug from a sporulating culture and mycelia were harvested from the colony centre and the peripheral hyphal zone as described [24]. For analyzing chitinase gene expression and extracellular endo- and exochitinase activities, *T. atroviride* was inoculated for 20 hours in minimal medium containing 1% glycerol as a carbon source. Mycelia were then harvested by filtration and transferred to media containing 1% N-acetyl-glucosamine (NAG) or 1% colloidal chitin. Mycelia and culture filtrates were harvested after 5, 14, and 24 hours from NAG-induced cultures and after 14, 24, 26, and 48 hours from chitin-induced cultures and stored at −20°C for enzyme assays or at −80°C for RNA extraction.

Plate confrontation assays with *Rhizoctonia solani* and *Botrytis cinerea* as hosts were performed as previously described [3], [25]. Pictures were captured from 24 hours until 14 days of growth. For RNA extraction, cultivations were performed on PDA plates covered with a sterile cellophane membrane. *Trichoderma* mycelium was harvested from the confrontation zone (5 mm of the peripheral area) before direct contact between the two fungi (5 mm distance), at direct contact, and after contact (5 mm overgrowth). Self-confrontations between the *Trichoderma* strains tested served as controls. Mycelia of the *T. atroviride* parental and mutant strains were frozen in liquid nitrogen and stored at −80°C.

### Enzyme assays

Enzymatic activities of culture supernatants were assayed as previously described [26] using the substrates p-nitrophenyl N-acetyl-β-D-glucosaminide for determination of N-acetyl-glucosaminidase and 4-nitrophenyl-β-D-N,N′,N″-triacetylchitotriose for determination of endochitinase activity. Enzyme activity was measured as U/ml (one unit is defined as the release of 1 µmol of nitrophenol per minute) relative to total intracellular protein assessed by Bradford assay (BioRad) and represented as enzyme activity in U/µg protein.

### Molecular techniques and mutant generation

In order to generate *T. atroviride* Δ*ste12* mutant strains, the DelsGate deletion construction methodology [27] was applied. ∼1 kb of the up- and downstream flanking non-coding regions of the *ste12* gene were amplified and recombined via phage attachment sites using BP clonase in a “Donor vector” (pDONR) containing the *hph* hygromycin B-phosphotransferase-encoding marker cassette. For amplification of the deletion vector, One Shot Omnimax 2 T1R *Escherichia coli* cells (Invitrogen, Carlsbad, CA) were used. The resulting *ste12* deletion vector pRAM was confirmed by PCR and DNA sequencing. In order to generate stable deletion mutants, the protoplast-based transformation method was applied as previously described [28]. Transformants were selected on PDA containing 200 µg/mL hygromycin B. Mitotically stable transformants were obtained by three rounds of single spore isolation and homologous integration of the deletion cassette was confirmed by PCR and Southern analysis [29] (Figure S1). Loss of *ste12* gene expression in the Δ*ste12* mutant was confirmed by RT-qPCR using the parental and the Δ*tmk1* strains as controls.

For complementation, a 5141-kb fragment bearing the *ste12* gene and its 5′ and 3′ regulatory regions was amplified from *T. atroviride* strain P1 by PCR using primers ste12-C-FW and ste12-C-RV (Table 1) and introduced into the Δ*ste12* mutant by co-transformation with plasmid p3SR2. Transformants were selected on acetamide-containing medium and purified by three rounds of single spore isolation.

**Table 1:** Oligonucleotides used in this study.

gene		Sequence (5′ to 3′)
*nag1*	FW	TGTCCTACAGCCTCTGCTGCAAAAGTTC
	RV	CATCTCCTCACAGACAAGCGGTGAAAG
*prb1*	FW	CGCACTGCTTCCTTCACCAACT
	RV	TTTCACTTCATCCTTCGCTCCA
*ech42*	FW	CGCAACTTCCAGCCTCAGAACC
	RV	TCAATACCATCGAAACCCCAGTCC
*sar1*	FW	CTCGACAATGCCGGAAAGACCA
	RV	TTGCCAAGGATGACAAAGGGG
*act1*	FW	GCACGGAATCGCTCGTTG
	RV	TTCTCCACCCCGCCAAGC
*ham7*	FW	GGCTCTTTACTCTTGCGTCGAC
	RV	CCGCCCAGCCATTGCGAAG
*nox1*	FW	CTCAAGATTCACACCTACCTCAC
	RV	GCAACAGAGGGACCACAGAAG
*hex1*	FW	AGGAGTCTTCCTTCATTGCCAAC
	RV	AGAACGAGGACACGGACGC
*tmk2*	FW	CAGATGCCCACTTCCAATCCTT
	RV	CAAAAGCTATTGATGTATGGTCTGTG
*tac6*	FW	CGGGACTTATGGTTTGGGCG
	RV	CGAACGGTCCAGATGCGG
*ham9*	FW	CACAAGGATGCCCAACAGATG
	RV	CCGAGGCATTGGGCTGGAG
*tef1*	FW	GGTACTGGTGAGTTCGAGGCTG
	RV	GGGCTCAATGGCGTCAATG
*ste12*	FW	CCTGTGGCCGACTGTTCAAG
	RV	ATTCTTCCTCTTCCTCGGCAG
*ste12-C*	FW	CACTGCACTGTATTCCGGCTCCC
	RV	CGATGGCAGCGAAGACAATGAG
*hph*	FW	GCCGATCTTAGCCAGACGAG
	RV	CTGCGGGCGATTTGTGTACG

### RT-qPCR analysis

Total RNA was extracted using the peqGOLD TriFast Solution (PeqLab, Erlangen, Germany). Frozen mycelia were homogenized using glass beads by grinding twice for 30 s in a RETSCH MM301 Ball Mill (Retsch, Haan, Germany). Isolated RNA was treated with Deoxyribonuclease I (Fermentas, St. Leon-Rot, Germany), purified with the RNeasy MinElute Cleanup Kit (Qiagen, Hilden, Germany) and reverse transcribed to cDNA using the Revert Aid H Minus First Strand cDNA Synthesis Kit (Fermentas, Vilnius, Lithuania). qPCR was carried out with the Mastercycler ep realplex real-time PCR system (Eppendorf, Hamburg, Germany) using iQ SYBR Green Supermix (Bio-Rad, Hercules, CA).

Relative gene transcript levels were quantified using *sar1*, *act1* or *tef1* as reference genes [30] and the expression ratios were calculated according to [31]. For samples derived from chitin- and N-acetyl-glucosamine-induced cultures, gene expression levels were normalized to basal expression levels from cultivations on 1% glycerol. All samples were analyzed in three independent experiments with three replicates in each run.

For expression profiling of genes putatively involved in CAT formation and hyphal fusion, parental and mutant strains were grown in stationary liquid cultures with MM containing 1% glucose, as described in [24] and peripheral and intra hyphal zones were harvested. mRNA levels were quantified and normalized to the corresponding signals of *tef1* as reference gene [30]. Statistical analysis was done by relative expression analysis with REST software using the Pair Wise Fixed Reallocation Randomisation Test [32].

Expression of *ste12* in *T. atroviride* during the mycoparasitic interaction with *R. solani* and during self-confrontation was analyzed by semi-quantitative RT-PCR. *tef1* gene expression was used as reference. All primer sequences are listed in Table 1.

### Biolog phenotype array analysis

The Biolog FF MicroPlate assay (Biolog Inc., Hayward, CA) which comprises 95 wells with different carbon-containing compounds and one well with water was used to investigate growth rates on pre-filled carbon sources. Conidia were collected from the *Trichoderma* strains (parental strain, *Δtmk1, Δste12*) and used as inoculums as described [33]. Inoculated microplates were incubated in darkness at 28°C, and OD750 readings determined after 18, 24, 42, 48, 66, 72, 96 and 168 hours using a microplate reader (Biolog), which measures the turbidity and reflects mycelia production on the tested substrate. All analyses were performed in triplicate. For comparative analysis, values from the 48 hours time point was used as this was within the linear growth phase of *T. atroviride* on the majority of carbon sources. Values were quantitatively illustrated using the Hierarchical Clustering Explorer 3 (HCE3) [34]. For all analyses the hierarchical clustering algorithm with average linkage and Euclidean distance measure was applied.

### Microscopic analyses

Microscopic analyses were mainly performed as described by [35]. Briefly, 500 µl PDA was spread onto glass slides, inoculated by placing *T. atroviride* and *R. solani* on opposite sides of the glass slides, and incubated on a moistened filter paper at 28°C in a Petri dish sealed with Parafilm. After 48–72 hours, the fungal hyphae were imaged with an inverted T300 microscope (Nikon, Tokyo, Japan). Images were captured with a Nikon DXM1200F digital camera and digitally processed using Photoshop CS3 (Adobe, San Jose, CA, US). For biomimetic assays, sterile nylon 66 fibers (approximate diameter 14 µm; Nilit, Migdal-Haemek, Israel) were placed on the media before inoculation. For analysis in liquid cultures, 1×10^6^ spores per ml in 250 µl of MM containing 1% glucose were spread onto the glass slide and pictures were taken at 24 h and 48 h as described above.

## Results

### Characteristics of the *T. atroviride* Ste12 homologue

The aim of this study was to characterize the role of the Ste12 transcription factor, which is assumed to act as a central component of the mycoparasitism-relevant signaling pathway involving the Tmk1 MAPK in *T. atroviride*.

**Figure 1:**
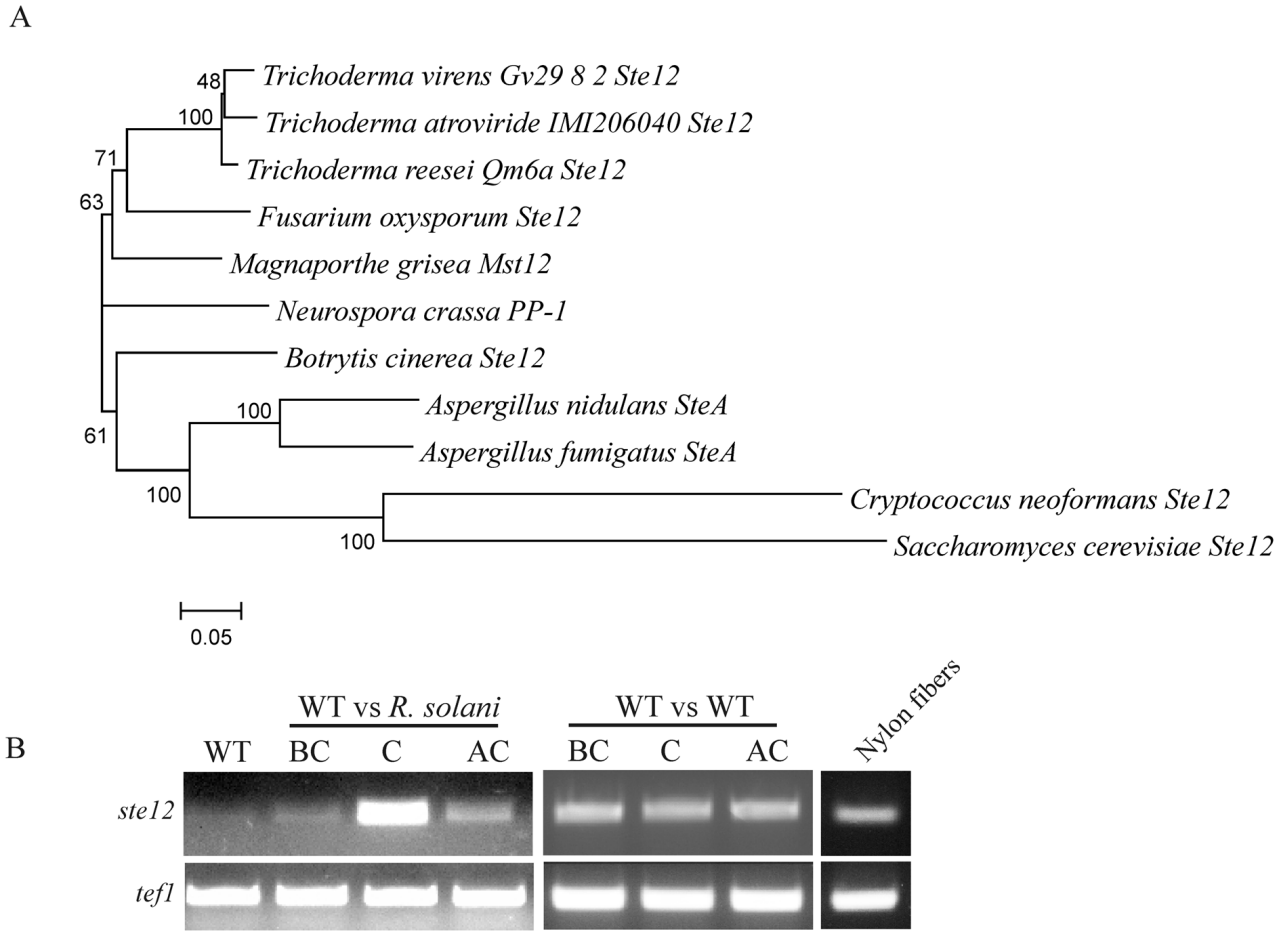
Phylogeny and transcriptional regulation of *T. atroviride ste12* (A) Phylogenetic analysis of Ste12-like proteins from various filamentous fungi. Ste12 orthologues identified in the genomes of the three *Trichoderma* species, *T. atroviride* (ID 29631), *Trichoderma virens* (ID 75179), and *Trichoderma reesei* (ID 36543), and *Fusarium oxysporum* Ste12 (ACM80357), *Magnaporthe oryzae* (*M. grisea*) Mst12 (AF432913), *Neurospora crassa* PP-1 (EAA28575), *Botrytis cinerea* Ste12 (ACJ06644.1), *Aspergillus nidulans* SteA (XP_659894.1), *Aspergillus fumigatus* SteA (EDP51368.1), *Cryptococcus neoformans* Ste12 (XP_776009.1), and *Saccharomyces cerevisiae* Ste12 (CAX80094.1) were aligned using ClustalX and the tree constructed using neighbor-joining algorithm with 1,000 bootstraps. (B) *ste12* transcription during growth of *T. atroviride* alone, in the mycoparasitic interaction with *R. solani* as host, in self-confrontation, and at contact with plain nylon fibers. *Trichoderma* mycelia were harvested from the interaction zone before direct contact (BC), at direct contact (C) and after contact (AC) between the two fungi. Products from RT-PCR reactions with primers targeting *ste12* and *tef1* (loading control) were separated by agarose gel electrophoresis.

The *T. atroviride ste12* gene (ID 29631;http://genome.jgi-psf.org/Triat2/Triat2.home.html) consists of an open reading frame of 2142-bp with two C-terminally located introns (60-bp and 63-bp) and is predicted to encode a protein of 672 amino acids. Ste12 displays considerable amino acid identity to the already functionally characterized Ste12 proteins of *Fusarium oxysporum* (ACM80357; 77%), *Magnaporthe oryzae* (*M. grisea*) (AF432913; 74%), *Neurospora crassa* (EAA28575; 65%), and *Aspergillus nidulans* (XP_659894; 60%) [12], [36]–[38]. Phylogenetic analysis showed Ste12 in a clade together with the orthologues from *Trichoderma virens* (ID 75179, http://genome.jgi-psf.org/TriviGv29_8_2/TriviGv29_8_2.home.html) and *Trichoderma reesei* (ID 36543, http://genome.jgi-psf.org/Trire2/Trire2.home.html), and with *F. oxysporum* Ste12 and *M. oryzae* Mst12 (Fig. 1). Similar to Ste12 proteins from other filamentous fungi, *T. atroviride* Ste12 contains an N-terminally located homeodomain-like (STE) motif (amino acids 54–163) which is presumed to be involved in DNA binding [39] and two distinct C-terminal C_2_H_2_ zinc finger domains (amino acids 546–582) which are distinguishing features of Ste12-like proteins of filamentous fungi [15].

For analyzing the expression of *ste12, T. atroviride* was either grown alone, in confrontation against *R. solani* as host, in self-confrontation, or in the presence of nylon fibers whose diameter resembles that of host hyphae. *ste12* was only moderately expressed when the fungus was grown alone, during self-confrontation, when coming into contact with plain nylon fibers, and at the pre-contact stage of the confrontation with the host (i.e. when *R. solani* was at a distance of 5 mm), whereas mRNA levels increased upon direct host contact (Fig. 1 B). The re-decline of *ste12* mRNA levels at the after contact stage, when *T. atroviride* had overgrown the host by 5 mm, indicates that this host-induced up-regulation of *ste12* expression is only transient and suggests a role of Ste12 in the regulation of mycoparasitism-relevant processes upon direct host contact.

In contrast to *Colletotrichum lindemuthianum* and *Botrytis cinerea*, for which alternative splicing of *ste12* has been described [40], [41], only full transcripts (data not shown) were found for *T. atroviride ste12*.

### Generation of *T. atroviride* ste12 deletion and complementation mutants

**Figure 2:**
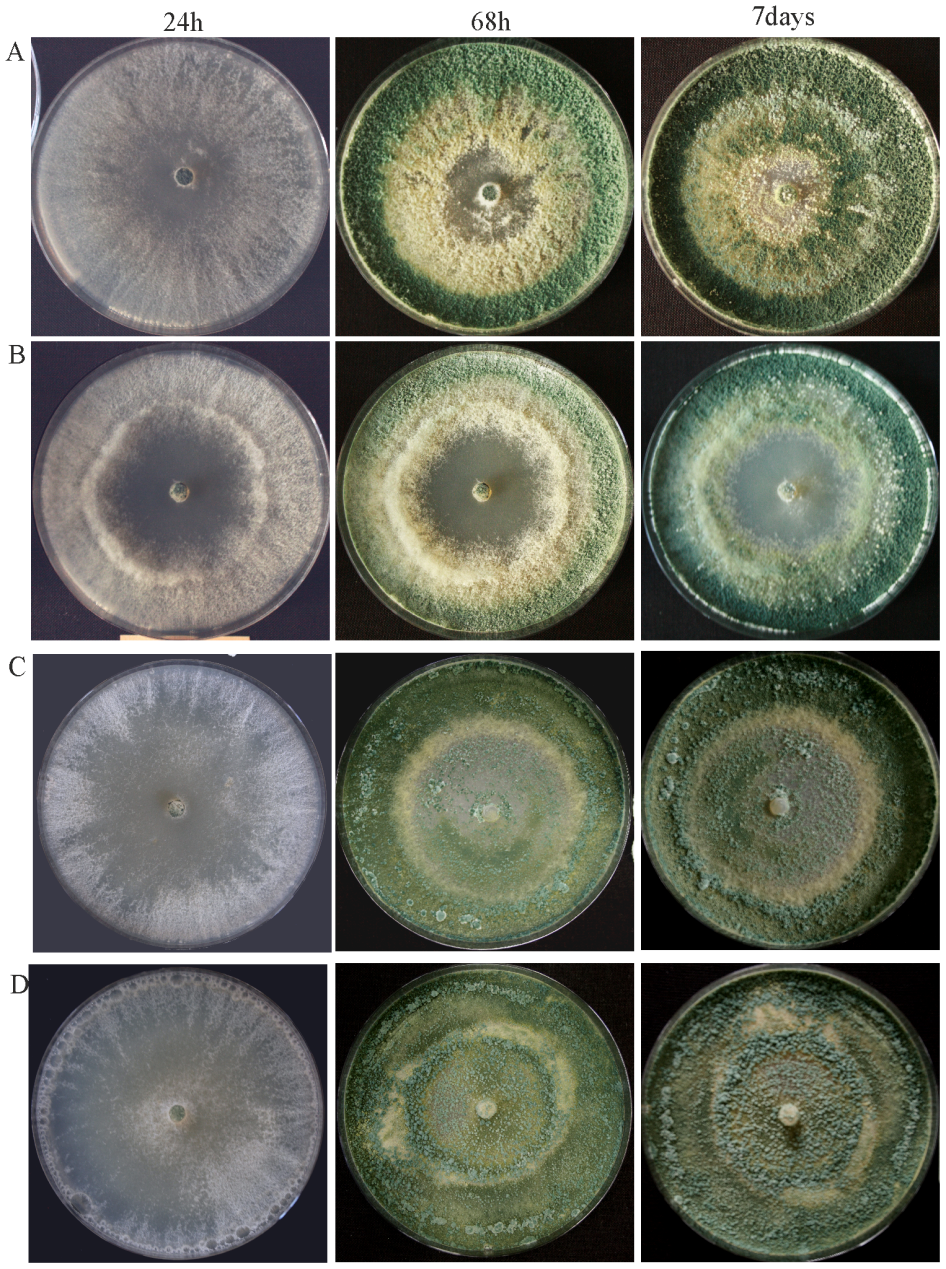
Colony morphology of Δ*ste12* (B) in comparison to the parental strain (A) and the complemented strains *ste12*-C1(C) and *ste12*-C2(D) upon growth on potato dextrose agar at 28°C for up to 7 days.

For functional characterization of *T. atroviride ste12*, we transformed the linearized *ste12* deletion vector into *T. atroviride* protoplasts. Although all of the resulting 20 transformants showed hygroymcin B-resistance, PCR- and Southern blot-based screening resulted in only one mutant with homologous integration and deletion of the *ste12* gene in a mitotically stable manner (Figure S1). Complemented strains were generated by introducing a 5141-kb fragment bearing the *ste12* gene and its 5′ and 3′ regulatory regions into the Δ*ste12* mutant. Two complemented transformants with an ectopically (*ste12*-C1) and homologously (*ste12*-C2) integrated *ste12* gene, respectively, were selected and included in a subset of experiments. The Δ*ste12* and complemented mutants exhibited growth rates similar to the parental strain on solid complete medium (PDA). However, deletion of *ste12* resulted in somewhat altered colony development with a reduced production of aerial hyphae in the colony centre and a delayed concentrical conidial ring formation. Whereas in the parental and both complemented strains the onset of conidiation starts in the middle of the colony, conidial maturation started from the subperipheral zone in Δ*ste12* (Fig. 2). Similar to the parental strain, conidiation in the Δ*ste12* mutant was light-dependent and the number of conidia produced by Δ*ste12* was similar to the parental strain (1.9±0.3 * 10^9^ and 1.2±0.2 * 10^9^, respectively).

### Ste12 impacts carbon utilization of *T. atroviride*

**Figure 3:**
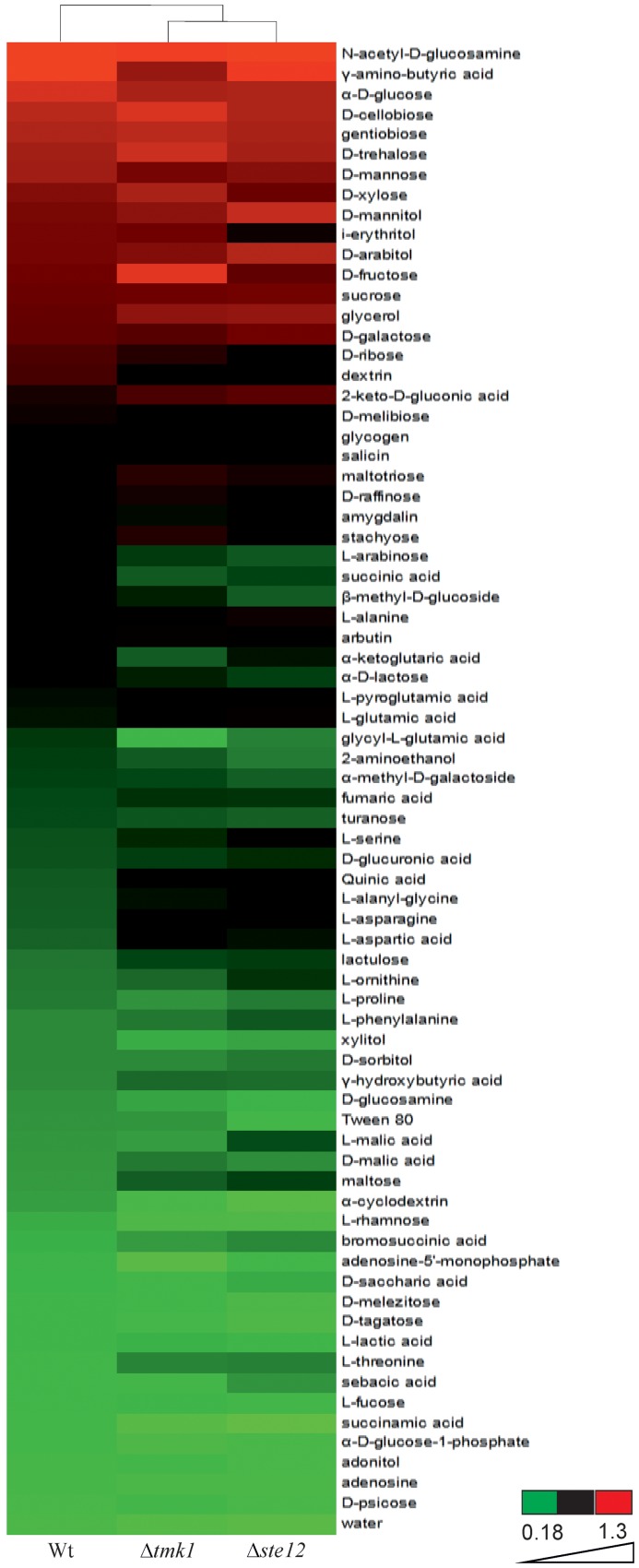
Comparative carbon source utilization profiles of the Δ*ste12* and Δ*tmk1* mutants and the parental strain (WT). Strains were grown on 95 carbon sources (FF-plates) using the Biolog Phenotype Microarray system. Hierarchical clustering results are displayed as coloured mosaics attached to a dendrogram according to growth (OD_750_) after 48 hours of incubation. All data points are the average of three replicates. The threshold cut-off for all conditions was set at 0.225 which corresponded to growth of the parental strain on the water control. Red boxes indicate maximal growth (OD_750_ 0.7–1.3), black boxes medium growth (OD_750_ 0.55–0.7), and green boxes weak growth (OD_750_ 0.2–0.55).

In order to get additional insights into the phenotypic consequences resulting from *ste12* deletion and to learn more on a putative involvement of Ste12 in the Tmk1 MAPK pathway, we performed comparative nutrient profiling of Δ*ste12* and Δ*tmk1* mutants. The carbon-source utilization profile of *T. atroviride* on 95 different carbon sources has been characterized previously resulting in four clusters [33]. Clusters I (comprising mainly monosaccharides and polyols, but also γ-amino-butyric acid and N-acetyl-D-glucosamine), II (several monosaccharides, some oligosaccharides and arylglucosides), and III (mainly disaccharides and oligosaccharides, arylglucosides and L-amino acids) contain carbon sources allowing fast, moderate and slow growth, respectively, while carbon sources only allowing very poor or no growth at all are contained in cluster IV (containing several L-amino acids, peptides, amines, TCA-intermediates, aliphatic organic acids). Analysis of growth of the Δ*ste12* and Δ*tmk1* mutants on the 95 carbon sources revealed similar carbon utilization profiles for both mutants which, however, significantly differed from the parental strain (Fig. 3). Amongst the cluster I-III carbon sources, D-mannose, D-ribose, dextrin, salicin, amygdalin, L-arabinose, succinic acid, and β-methyl-D-glucoside only allowed reduced growth of both mutants compared to the parental strain. On the other hand, both, Δ*ste12* and Δ*tmk1*, exhibited enhanced growth on 2-keto-D-gluconic acid, glycerol, maltotriose, quinic acid and the amino acids and amino acid derivatives L-glutamic acid, L-asparagine, L-aspartic acid, L-threonine, L-pyroglutamic acid, and L-alanyl-glycine. In addition to this congruent behavior, also differences in the nutritional profiles between Δ*ste12* and Δ*tmk1* mutants were evident. While on i-erythritol, the Δ*tmk1* mutant showed similar growth than the parental strain, the Δ*ste12* mutant showed significantly reduced growth. Vice versa, deletion of *tmk1*, but not *ste12*, resulted in reduced growth on γ-amino-butyric acid which, together with N-acetyl-D-glucosamine, was the best carbon source for the *T. atroviride* parental strain. On the other hand, the Δ*tmk1* mutant exhibited better growth compared to the *ste12* deletion mutant and the parental strain on the monosaccharides D-trehalose, D-xylose, and D-fructose, the disaccharide cellobiose and the tetrasaccharide stachyose, while Δ*ste12* grew better on the polyols D-mannitol and D-arabitol.

Summarizing, vegetative growth and conidiation of *T. atroviride* on rich medium remained largely unaffected by deletion of the *ste12* gene. Nutrient profiling, however, revealed effects of Ste12 and Tmk1 on the utilization of certain carbohydrates and amino acids as carbon sources. Consequently, we conclude that the regulation of growth by Tmk1 is carbon source-dependent and only partially mediated by Ste12.

### Ste12 impacts hyphal morphology and negative hyphal autotropism

**Figure 4:**
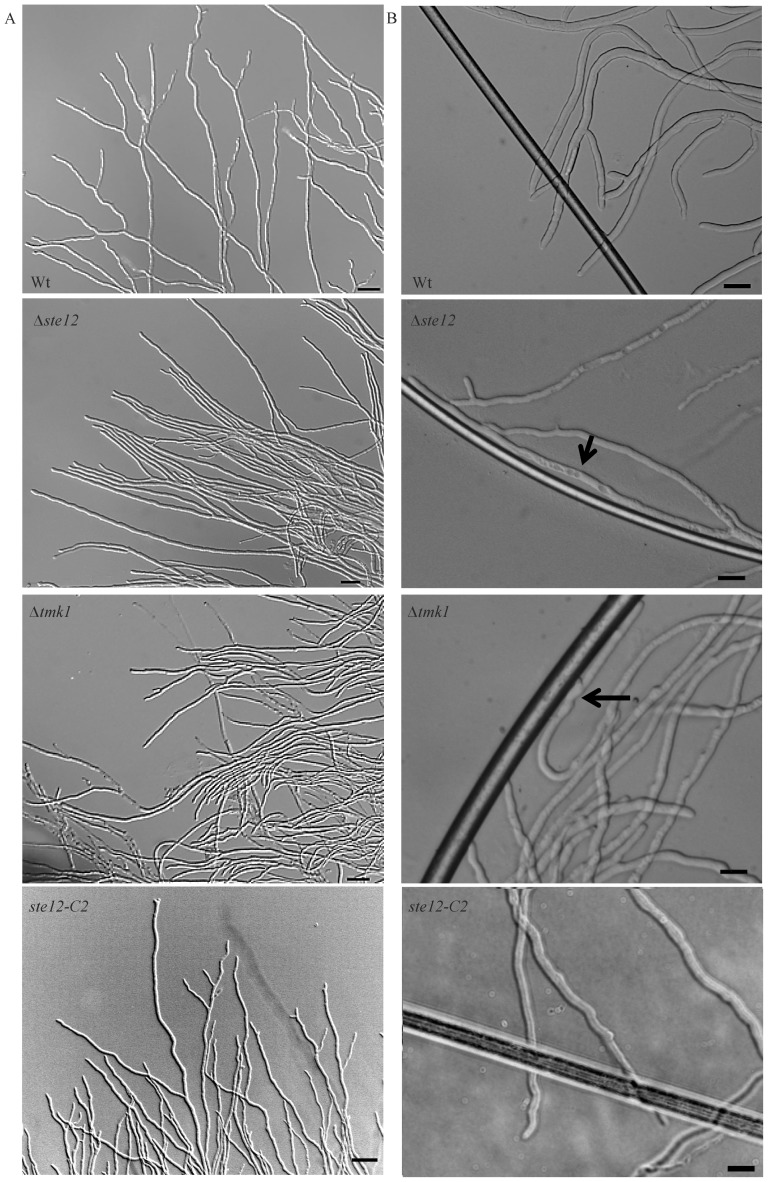
Phenotypes of the Δ*ste12* and Δ*tmk1* mutants compared to the parental strain (WT) and the complemented strain *ste12*-C2 upon growth on potato dextrose agar (PDA). (A) Hyphae of Δ*ste12* and Δ*tmk1* mutants attached and formed hyphal aggregates in the colony periphery whereas the parental and the complemented strain showed hyphal avoidance. (B) Light microscopy of hyphae of the Δ*ste12* and Δ*tmk1* mutants, the parental strain, and the complemented strain *ste12*-C2 upon growth on PDA with plain nylon fibers (approximate diameter 14 µm). Attachment to and growth along the fibers of hyphae of Δ*ste12* and Δ*tmk1* mutants is marked with arrows.

During normal mycelial growth, hyphal tips are engaged in environmental sensing and usually avoid each other (negative autotropism) allowing the fungus to explore and exploit the available substrate whereas sub-apical hyphal parts generate new branches (reviewed in [42]). Microscopic analyses of Δ*ste12* mycelia from the colony periphery revealed long hyphae with only few branches which aberrantly clustered by growing alongside each other thereby resulting in compact hyphal bundles (Fig. 4 A). This loss of hyphal avoidance was also apparent in Δ*tmk1* mutants but not in the parental strain and the complemented strain *ste12*-C2 which at the colony periphery formed typical branched hyphae that grew away from their neighbours.

To explore whether the aberrant hyphal aggregation caused by deletion of *ste12* or *tmk1* is due to a de-regulated sensing mechanism, the biomimetic system [43] was used. Frequent attachment to and growth alongside uncoated nylon fibers was observed in Δ*ste12*, similar to the behaviour of Δ*tmk1* mutants but contrasting the parental and the complemented strain (Fig. 4 B). Interestingly, this attachment was accompanied by enhanced branching of the Δ*ste12* mutant's hyphae, a reaction normally displayed by the *T. atroviride* wild-type in response to the presence of a host fungus [35].

We conclude that the role of the Tmk1 MAPK in suppressing attachment to and coiling around own hyphae and foreign hyphal-like structures in the absence of respective cues is mediated by the Ste12 transcription factor.

### Ste12 impacts mycoparasitic overgrowth and host lysis

Our previous analyses of Δ*tmk1* mutants revealed reduced mycoparasitic activity and altered host specificity as Δ*tmk1* mutants still could parasitize and at least partially lyse *R. solani*, whereas they completely lost the ability to antagonize *B. cinerea* [17].

**Figure 5:**
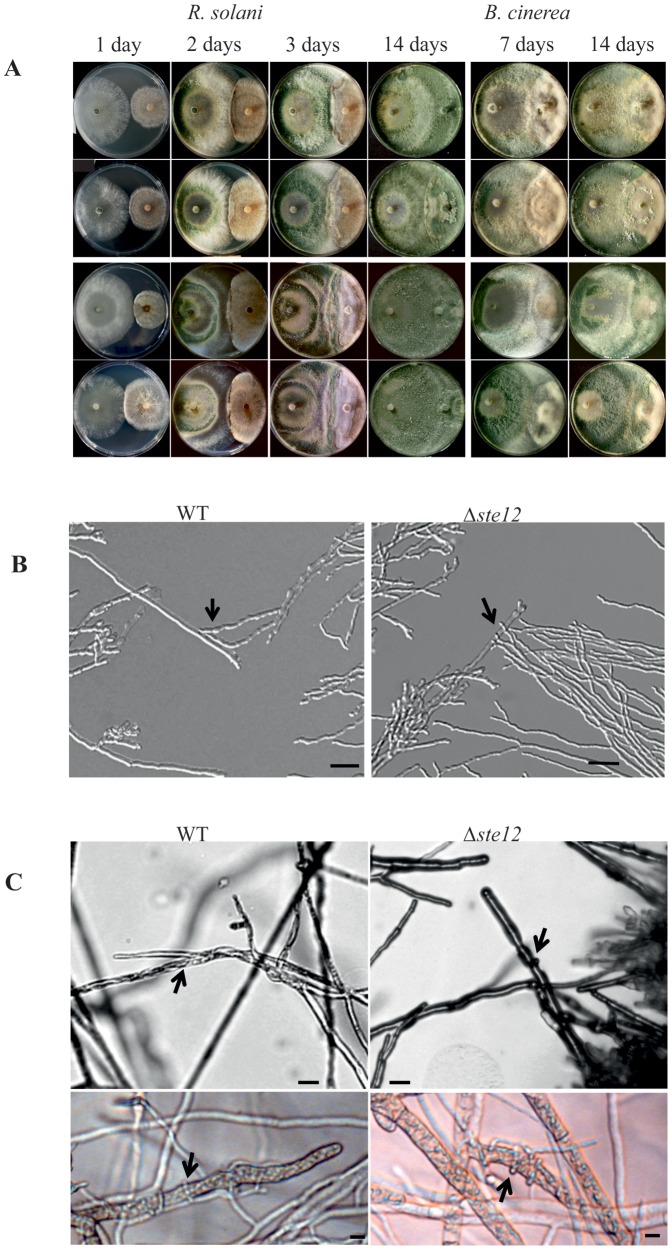
Mycoparasitic activity of the Δ*ste12* mutant against *R. solani* and *B. cinerea* as hosts. (A) Plate confrontation assays of the Δ*ste12* mutant (second panel), the parental strain (upper panel) and the complemented strains *ste12*-C1 (third panel) and *ste12*-C2 (fourth panel) against host fungi. Pictures were taken 1, 2, 3, and 14 days (*R. solani*) and 7 and 14 days (*B. cinerea*) after inoculation of the two fungi on opposite sides of the plate. (B) Microscopic analyses of the confrontation zone between *T. atroviride* (right side) and *R. solani* (left side). The Δ*ste12* mutant approaches the host as aggregated hyphae with only single hyphae attaching to *Rhizoctonia*. Attachments to host hyphae are marked by arrows. The scale bar represents 50 µm. (C) Attachment to and coiling around host hyphae. Despite the inability of the Δ*ste12* mutant to fully overgrow and parasitize *B. cinerea*, the mutant shows the typical mycoparasitism-associated coiling response.

In order to assess whether Tmk1 regulates the mycoparasitic activity of *T. atroviride* by employing the Ste12 transcription factor and whether the observed aberrant self-attachment of Δ*ste12* impacts the mycoparasitic interaction with a living host fungus, plate confrontation assays with *R. solani* and *B. cinerea* as fungal hosts were performed. During the early phases of the interaction with *R. solani*, i.e. growth towards the host fungus and establishment of direct contact, the Δ*ste12* mutant behaved similar as the parental strain (Fig. 5 A). After 14 days however, *R. solani* hyphae were completely lysed by the parental and the complemented strain while only incompletely lysed by the Δ*ste12* mutant. A reduction in the mycoparasitic attack and host lysing abilities of the mutant was also evident against *B. cinerea*. While the parental and the complemented strain steadily overgrew the host, *ste12* deletion resulted in a halt shortly after establishment of contact between the two fungi which could hardly be overcome by the Δ*ste12* mutant even under prolonged incubation times (Fig. 5 A).

Imaging of the confrontation zone between *T. atroviride* and *R. solani* revealed typical growth of the parental strain towards the host followed by attachment to and growth alongside host hyphae (Fig. 5 B). Whereas the parental strain grew as well separated hyphae, the Δ*ste12* mutant approached the host primarily in the form of hyphal bundles with only single hyphae attaching to *Rhizoctonia*. Microscopic examination of individual hyphae of the Δ*ste12* mutant during the interaction with either *R. solani* or *B. cinerea* revealed typical mycoparasitism-associated morphological changes, i.e. hyphal attachment to and coiling around the host (Fig. 5 C). From these results we conclude that Ste12 affects mycoparasitic overgrowth and host lysis in a host-specific manner although attachment to and coiling around the host hyphae is unaltered upon *ste12* deletion.

### Ste12 affects the expression of cell wall-degrading enzymes

The lysis of the host's cell wall is a key process in mycoparasitism [4]. We therefore tested whether Ste12 mediates the Tmk1-dependent regulation of chitinase gene expression. As reported previously, Δ*tmk1* mutants attained higher *nag1* (N-acetyl-D-glucosamidase I-encoding) and *ech42* (endochitinase 42-encoding) transcript levels and enhanced extracellular N-acetyl-glucosaminidase (NAGase) and endochitinase activities under chitinase-inducing conditions [17].

**Figure 6:**
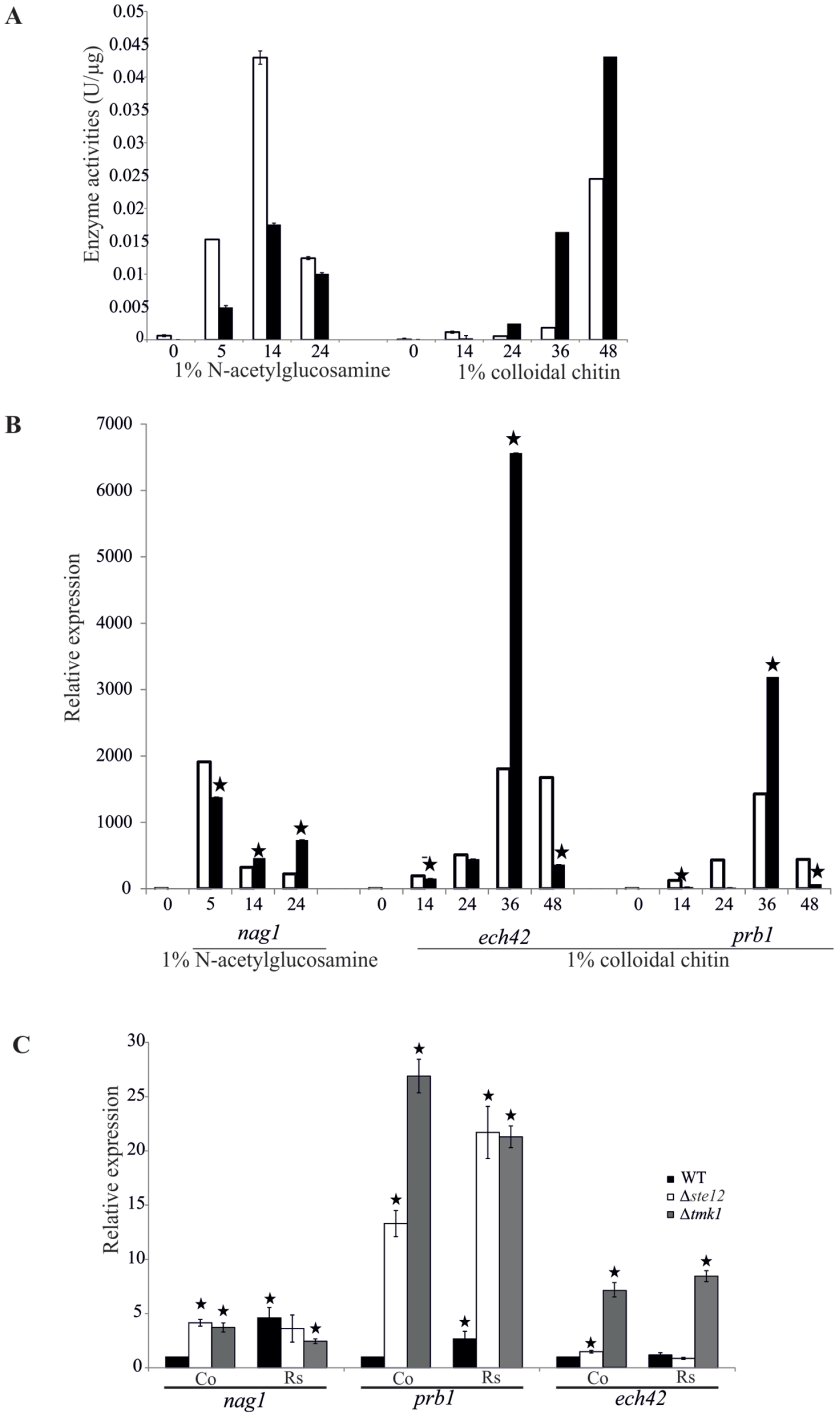
Impact of Ste12 on the expression of mycoparasitism-related cell wall-degrading enzymes. (A) Extracellular N-acetyl-glucosaminidase (NAGase) and endochitinase activities in the Δ*ste12* mutant (black bars) and the parental strain (white bars). After pre-cultivation on 1% glycerol, mycelial biomass was transferred to 1% N-acetyl-glucosamine-containing media for inducing NAGases and to 1% colloidal chitin-containing media for induction of endochitinases. Culture filtrates were harvested at the indicated time points and determined enzyme activities related to intracellular total protein. (B) Relative transcription ratios of the chitinase-encoding *nag1* and *ech42* genes and the *prb1* protease-encoding gene in the Δ*ste12* mutant (black bars) and the parental strain (white bars). RT-qPCR analyses were performed 5, 14, and 24 hours after transfer to N-acetyl-glucosamine (*nag1*) and 14, 24, 36, and 48 hours after transfer to colloidal chitin (*ech42, prb1*) using *act1* as reference gene. Un-induced samples of the parental strain harvested after pre-cultivation in glycerol-containing media were arbitrarily assigned the factor 1. Asterisks indicate significantly different (p≤0.05; calculated by REST software) transcription ratios of the mutant compared to the parental strain. (C) Relative transcription ratios of *nag1*, *ech42* and *prb1* in Δ*ste12* (white bars) and Δ*tmk1* (grey bars) mutants and the parental strain (black bars) upon direct confrontation with *R. solani*. Samples were collected from a control (co) where *Trichoderma* was confronted with itself and from the early overgrowth stage in the confrontation with *R. solani* (Rs) and subject to RT-qPCR using *sar1* as reference gene. The control sample of the parental strain was arbitrarily assigned the factor 1 and those samples which show significant differences (p≤0.05; calculated by REST software) to this control are marked with an asterisk. Results shown are means ±SD (n = 3).

While secreted NAGase activities in N-acetyl-glucosamine-induced cultures were decreased upon *ste12* deletion at all time points tested, the Δ*ste12* mutant showed elevated extracellular endochitinase activities compared to the parental strain upon induction with colloidal chitin (Fig. 6 A). Further analysis at the transcript level confirmed the enhanced transcription of the *ech42* gene in the Δ*ste12* mutant after cultivation on colloidal chitin for 36 hours and, unexpectedly, also revealed enhanced *nag1* mRNA levels compared to the parental strain upon cultivation in the presence of N-acetyl-glucosamine for 14 and 24 hours (Fig. 6 B). Similar to *ech42*, the *prb1* gene, which encodes a subtilisin-like serine protease whose over-expression has been shown to improve the biocontrol activity of *T. atroviride* [44], can be induced by chitin. *prb1* expression was highest after 36 hours of cultivation in chitin-containing media in both the Δ*ste12* mutant and the parental strain with *prb1* mRNA levels being ∼2-fold enhanced in the mutant.

Based on the findings that Ste12 negatively regulates the expression of the cell wall-degrading enzymes tested but positively affects the mycoparasitic activity of *T. atroviride* against *R. solani* and *B. cinerea*, we were interested in analyzing the expression of the mycoparasitism-relevant *ech42*, *nag1* and *prb1* genes in direct confrontation assays. To this end, mycelia from the parental strain and the Δ*ste12* and Δ*tmk1* mutants during direct interaction with *R. solani* were harvested at the early overgrowth stage which corresponded to the stage with the most significant differences between the Δ*ste12* mutant and the parental strain in the mycoparasitism assays (Fig. 5 A). While in the parental strain all three tested genes were significantly induced during overgrowth of *R. solani* compared to the self confrontation control, only *prb1* showed a host-induced expression pattern in the Δ*ste12* mutant with mRNA levels exceeding those of the parental strain by several-fold. Expression of *nag1* was elevated in both, the Δ*ste12* and the Δ*tmk1* mutants, although in a host-independent manner, i.e. also in the self confrontation control. Similarly, *ech42* gene transcription was independent from host-derived signals in both mutants with *ech42* mRNA levels in the Δ*tmk1* mutant significantly exceeding those of the parental strain and of the Δ*ste12* mutant (Fig. 6 C).

These results suggest that Ste12 and Tmk1 negatively regulate the expression of genes important for host cell wall degradation and host lysis in *T. atroviride*.

### Ste12 mediates the influence of Tmk1 on CAT formation and hyphal fusion

Vegetative hyphal fusion is important for the development of a mycelial network during colony development in many filamentous fungi. In addition to fusion of hyphae within a mature colony, germlings of *N. crassa* recognize each other shortly after conidial germination and can fuse via specialized structures, the conidial anastomosis tubes (CATs) [45]. In *N. crassa* and *F. oxysporum*, the Fus3/Kss1 homologues Mak-2 and Fmk1, respectively, are required for both CAT and hyphal fusion during vegetative growth [21], [46].

**Figure 7:**
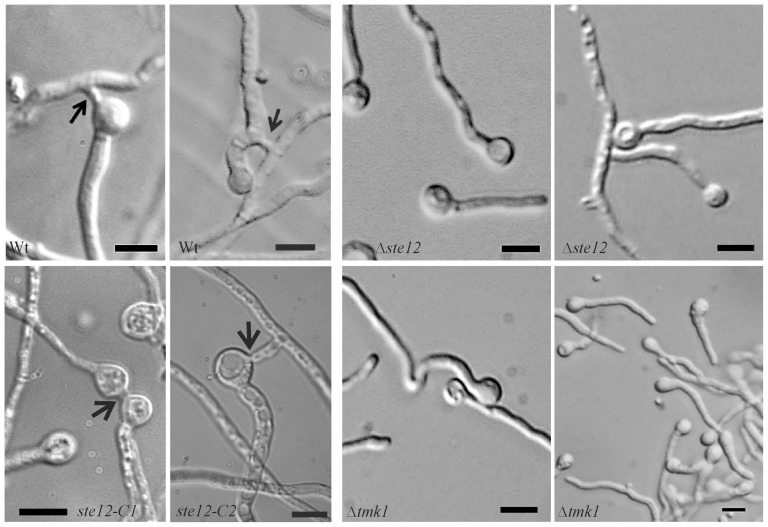
Analysis of conidial anastomosis tube (CAT) formation and hyphal fusion in Δ*ste12* and Δ*tmk1* mutants. Microscopic analyses of germlings of Δ*ste12* and Δ*tmk1* mutants, the parental strain and the complemented strains *ste12*-C1 and *ste12*-C2 16 hours after inoculation of conidia in potato dextrose broth. CATs and fusion bridges between germ tubes of the parental strain are marked with arrows. The scale bar represents 10 µm.

Despite the fact that *T. atroviride* Δ*ste12* and Δ*tmk1* mutants lost negative hyphal autotropism in the colony periphery which resulted in the observed hyphal aggregation, we were not able to detect distinct fusions between aggregated hyphae. To further analyze a putative role of Tmk1 and Ste12 in regulating fusion processes, the behavior of Δ*ste12* and Δ*tmk1* germlings was assessed microscopically. Conidial anastomosis tubes as well as fusion bridges between germ tubes were frequently observed in the parental and the complemented strain (Fig. 7). In contrast, CATs could not be detected in the Δ*ste12* and Δ*tmk1* mutants and also fusions between germ tubes were only rarely observed despite frequent contacts between the germlings. It is worth mentioning that Δ*tmk1* conidia showed delayed germ tube formation and extensive aggregation of Δ*tmk1* germlings occurred (Fig. 7). Recent studies in the fungal model *N. crassa* led to the identification of target genes being required for cell fusion which are under control of the Mak-2 MAPK and the Ste12 homolog PP-1 [45]. To further substantiate our above findings of Tmk1 and Ste12 playing key roles in fusion processes in *T. atroviride*, mycelia of Δ*ste12* and Δ*tmk1* mutants and the parental strain were harvested from the internal as well as peripheral zones of the fungal colony. For gene expression analyses, the respective *T. atroviride* orthologues (Ta300768, *tmk2*, *hex1*, Ta302802, Ta294940) of the *N. crassa* fusion genes *ham-7* (encoding a GPI-anchored protein required for activation of the cell wall integrity MAPK MAK-1), *mak-1* (MAPK), *hex-1* (involved in septal plugging), *nox-1* (NADPH oxidase), and *ham-9* (pleckstrin domain protein) were identified in the *T. atroviride* genome database (http://genome.jgi-psf.org/Triat2/Triat2.home.html) by BLAST searches. Moreover, the glycosyl-hydrolase 18 (GH18) subgroup C chitinase-encoding gene *tac6* (Ta348129), which plays a role in hyphal network formation in *T. atroviride* [24], was included in the study.

**Figure 8:**
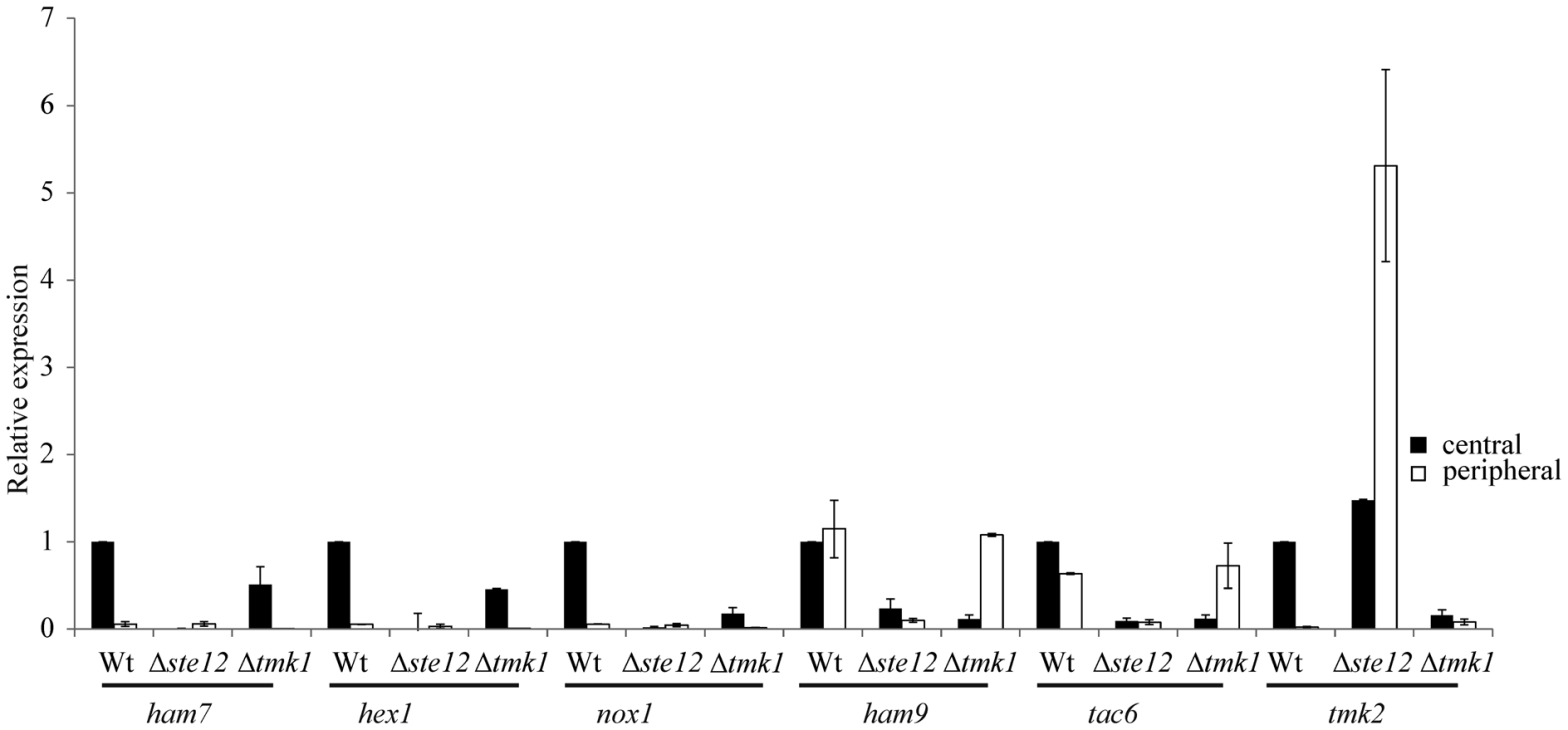
Impact of Ste12 and Tmk1 on the transcription of genes with a putative role in hyphal fusion. Relative transcription ratios of the putative fusion genes (see text) in the colony center and the peripheral zones of colonies of the Δ*ste12* and Δ*tmk1* mutants and the parental strain were determined by RT-qPCR using *tef1* as reference gene. The sample from the central region of parental strain colonies was arbitrarily assigned the factor 1. Results shown are means ±SD (n = 3).

Of the genes tested, Ta300768/*ham-7*, *hex1*, Ta302802/*nox-1*, *tmk2*, and *tac6* showed a significantly higher transcription in the center than at the peripheral zone of *T. atroviride* colonies. This expression pattern would be indicative of a role of these genes in hyphal fusion which predominantly takes place in the colony center (Figure 8). In accordance with *tmk1* and *ste12* deletion resulting in a loss of cell fusion, mRNA levels of Ta300768/*ham-7*, *hex1*, Ta302802/*nox-1*, and *tac6* were reduced in both the center and the periphery of Δ*ste12* and Δ*tmk1* colonies compared to the parental strain colony center. Ta294940/*ham9* was found to be similarly transcribed throughout the wild-type colony and the Δ*tmk1* peripheral zone but showed heavily reduced expression in Δ*ste12* colonies. A completely different picture was obtained for *tmk2*, which encodes the *T. atroviride* homolog of the cell wall integrity pathway MAPK Slt2 of *S. cerevisiae* and Mak-1 of *N. crassa*. While deletion of *tmk1* was found to result in significantly reduced *tmk2* transcription, Δ*ste12* mutants showed enhanced *tmk2* mRNA levels especially in the peripheral zone of the colony (Figure 8). These results show that expression of the cell wall integrity MAPK Tmk2 in *T. atroviride* is positively regulated by Tmk1 but negatively affected by Ste12 under the conditions tested and suggest that the repressing effect of Ste12 is mediated by upstream components other than Tmk1.

## Discussion

The genus *Trichoderma* comprises potent antagonists with many species being able to parasitize and kill other fungi [4]. As a prerequisite for this mycoparasitic lifestyle, *Trichoderma* has to possess appropriate receptors and intracellular signaling pathways for sensing and integrating signals derived from host fungi. Orthologues of yeast Fus3 (mating pathway) and Kss1 (filamentous growth pathway) MAPKs are multifunctional pathogenicity factors required for virulence of biologically and taxonomically diverse fungi [20]. However, although the pathogenicity MAPK (Pmk) pathway modules have been conserved throughout evolution [47] the output responses are species-specific and it is still not clear how exactly Pmk-type MAPKs regulate fungal virulence. In the foliar plant pathogen *M. oryzae*, the Pmk1 cascade targets the transcription factor Mst12, an ortholog of yeast Ste12. Similar to *pmk1* mutants, *mst12* mutants fail to penetrate the plant surface and are compromised in infectious growth although Mst12, in contrast to Pmk1, is dispensable for appressorium formation in *M. oryzae* [38], [48]. A comparable situation has been reported for non appressorium-forming fungi such as *F. oxysporum* where invasive growth is regulated by the Fmk1 MAPK and the transcription factor Ste12 [19].

Here, we have characterized Ste12 from the mycoparasitic fungus *T. atroviride* by assessing its role in mediating vegetative growth, colony development and mycoparasitism-related functions. The structure of *T. atroviride* Ste12 is similar to Ste12 proteins from other filamentous fungi and contains the typical homeodomain-like STE domain and two C_2_H_2_ zinc finger motifs. In *S. cerevisiae* Ste12, the homeodomain is required for binding to the regulatory protein Dig2 and the cofactor Tec1, whereas the central and C-terminal regions are involved in homodimerisation and binding to the negative regulator Dig1 and the Mcm1 transcription factor (reviewed by [11], [47]). Whereas we found a conserved Mcm1 orthologue in the *T. atroviride* genome (ID 223702), *T. atroviride* does not encode Dig1 and Dig2 like proteins - a situation similar to other filamentous fungi [47]. An orthologue of Tec1, which upon heterodimerisation with Ste12 induces filamentous growth under nutrient limiting conditions in *S. cerevisiae* [10], is encoded in the genome of the filamentous pathogen *A. fumigatus* but could neither be detected in plant pathogenic ascomycetes [47] nor in our study in the mycoparasite *T. atroviride*. The homeodomain of Ste12 has previously been shown to be required for DNA binding in *Cryptococcus neoformans* and *Colletotrichum lindemuthianum* [40], [49] and to be essential for all characterized functions of the *N. crassa* Ste12 homolog PP-1 [45]. The C-terminal zinc finger motifs, which are characteristic for Ste12 proteins of filamentous fungi but missing in *Saccharomycotina*, have recently been reported as dispensable in *N. crassa* PP-1, they are, however, together with the homeodomain required for Mst12-mediated virulence in *M. oryzae* [38], [50].

*ste12* mRNA levels were transiently up-regulated in *T. atroviride* at the stage of direct contact with the host fungus *R. solani*. A similar host-induced regulation of *ste12* expression occurs in *F. oxysporum* where transcription of *fost12* is up-regulated during the early stages of host plant colonization [36].

**Figure 9:**
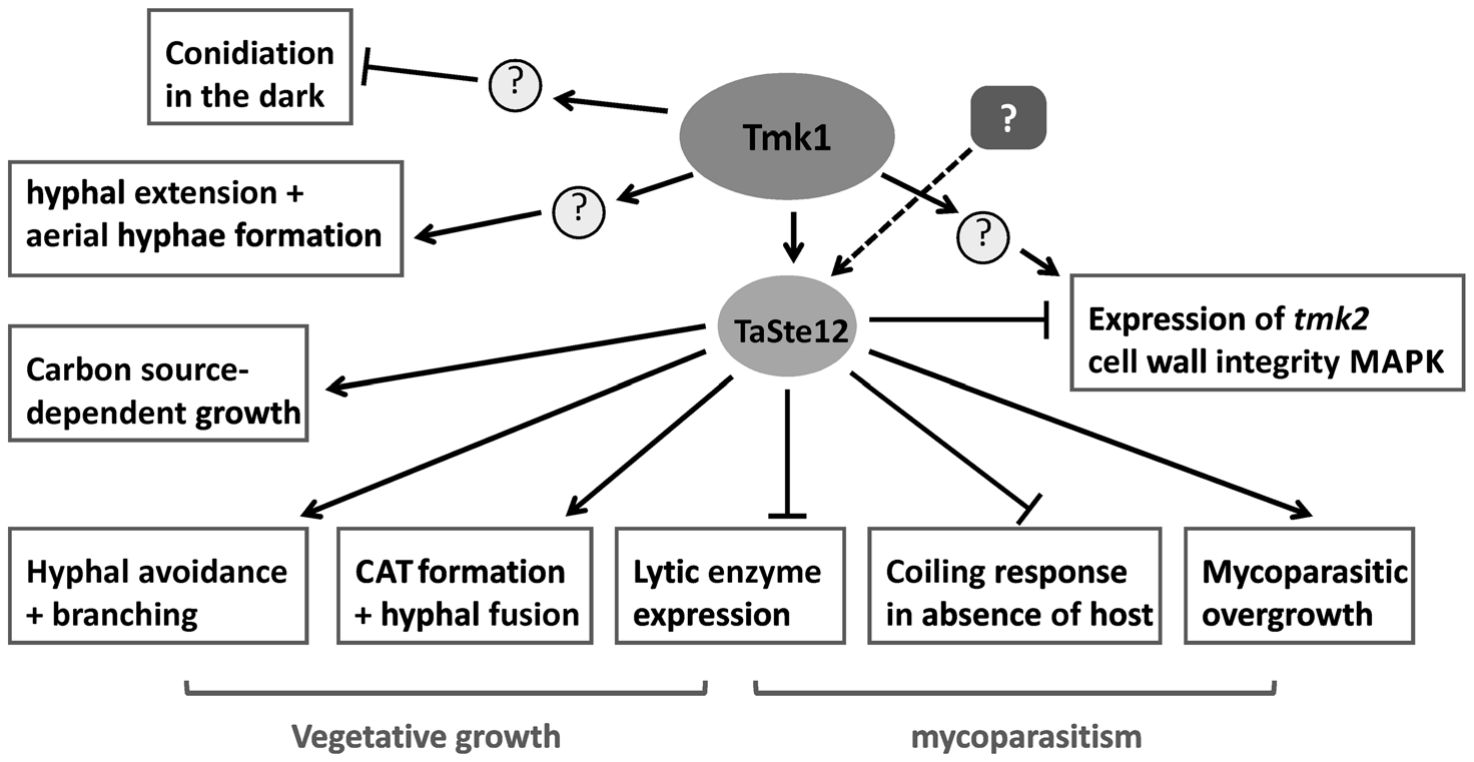
Model of the role of Ste12 as down-stream target of the Tmk1 MAP kinase pathway and as target of additional, still unknown pathways.

To substantiate a putative role of Ste12 in the mediation of mycoparasitism-relevant processes and as a functional target of the Tmk1 MAP kinase, the *ste12* gene was deleted in *T. atroviride*. Comparative analyses of the phenotypes of Δ*ste12* and Δ*tmk1* mutants revealed that several of the Tmk1 MAPK outputs are mediated by Ste12 (Fig. 9). The Δ*ste12* and Δ*tmk1* mutants shared defects in hyphal avoidance and anastomosis and showed similar carbon-source utilization profiles and alterations in mycoparasitism-related processes, with the latter, however, being more pronounced upon *tmk1* deletion. Also differences between Δ*ste12* and Δ*tmk1* mutants were found which suggests that additional transcription factors other than Ste12 are targeted by Tmk1. As reported previously, Δ*tmk1* mutants form “flat” colonies with only few aerial hyphae, show reduced hyphal growth rates on PDA and sporulate light-independently [17], [23]. In the present study we further found that *tmk1* deletion resulted in a delay in germ tube elongation, similar to what has been reported for *N. crassa mak-2* deletion strains and which indicates that a functional MAPK is required for optimal apical hyphal extension [46]. In contrast, *T. atroviride* Ste12 is dispensable for hyphal extension and is not involved in mediating the repressing effect of Tmk1 on conidiation in the dark; furthermore, it plays only a minor role in aerial hyphae formation.

Δ*ste12* and Δ*tmk1* mutants showed in large parts overlapping carbon source utilization profiles which, however, significantly differed from that of the parental strain. Regulation of primary metabolic pathways by the Pmk1 MAPK has previously been reported for *M. oryzae* [51], [52] and in *C. neoformans*, the mediator protein Ssn8, which in *S. cerevisiae* is involved in carbon utilization, acts downstream of the Cpk1 MAPK and the Ste12 transcription factor [53]. Interestingly, both Δ*ste12* and Δ*tmk1* mutants showed reduced growth on γ-amino-butyric acid (GABA) with a more severe growth reduction resulting from *tmk1* gene deletion. GABA metabolism is required for full pathogenicity in the wheat pathogen *Stagnospora nodorum* [54]. A similar situation may apply to *T. atroviride* where deletion of *ste12* or *tmk1* results in impaired mycoparasitism being more pronounced in Δ*tmk1* than Δ*ste12* mutants.

In the fungal model *N. crassa*, hyphal fusion occurs between germlings during colony establishment and between hyphae in subapical parts of mature colonies. Cell fusion in germinating conidia is associated with the production of specialized fusion structures, the conidial anastomosis tubes (CATs) [55]. *Neurospora* strains carrying deletions of Fus3 MAPK pathway components such as *nrc-1*, *mek-2*, *mak-2* or *pp-1* are defective in cell fusion and are female sterile [45], [46], [56], [57].

Phenotypic analyses of *T. atroviride* Δ*ste12* and Δ*tmk1* mutants revealed a loss of CAT formation and a severe reduction of hyphal fusion events, whereas CATs and fusion bridges were frequently observed in the parental strain. Interestingly, to the best of our knowledge, conidial anastomosis tube formation has never been shown before in *Trichoderma* although there are several reports on inter- and intra-strain anastomosis of vegetative hyphae or protoplast fusion mainly with the aim to obtain new genetic combinations with improved biocontrol activities (e.g. [58], [59]). Our results suggest that the Tmk1 MAPK pathway regulates CAT formation and hyphal fusion in *T. atroviride* by employing the Ste12 transcription factor. This is similar to the situation in *N. crassa*, but different to *F. oxysporum* where the essential role of the Fmk1 MAPK in vegetative hyphal fusion is not mediated by Ste12 [19].

Formation of an interconnected mycelial network by hyphal fusions is important for communication and translocation of water and nutrients within a filamentous fungus' colony [60]. Loss of or reductions in vegetative hyphal fusion events hence are supposed to result in a decrease in mycelial interconnections and a concomitant reduced nutrient transport from the colony periphery, where fresh medium is available, to the colony center, where nutrients are increasingly exploited. It may be speculated that the altered growth phenotype of *T. atroviride* Δ*ste12* and Δ*tmk1* mutants, i.e. the formation of aggregated hyphal bundles, which typically are formed by fungi on an exhausted substrate, is due to the reduced abilities of the mutants to anastomose.

Transcriptional analysis of conserved *T. atroviride* orthologues being targets of the PP-1 transcription factor in the *N. crassa* cell fusion pathway further supported the fusion defects of the Δ*ste12* mutant. Similar to *N. crassa* PP-1 [45], Ste12 showed an activating role on the expression of putative fusion genes such as Ta300768 (*ham7*), Ta294940 (*ham9*), Ta298536 (*hex1*), and Ta302802 (*nox1*) in *T. atroviride*. However, there were remarkable differences between the two fungi concerning the role of Ste12 in regulating the expression of the cell wall integrity MAPK-encoding gene. Whereas *mak-1* expression is reduced in *N. crassa* Δ*pp-1* strains [45], the *T. atroviride* Δ*ste12* mutant showed elevated *tmk2* mRNA levels. In the peripheral zones of the colonies, *ste12* or *tmk1* deletion resulted in 256-fold and 4-fold enhanced *tmk2* transcript levels, respectively, compared to the parental strain whereas in the colony centre only the Δ*ste12* but not the Δ*tmk1* mutant over-expressed *tmk2*. *T. virens tmkB* mutants, missing the TmkB homologue of the yeast cell wall integrity MAPK Slt2, showed increased sensitivity to cell wall-degrading enzymes and had cell wall integrity defects [61]. It may be speculated that the elevated transcription of *tmk2* in the periphery of *T. atroviride* Δ*ste12* and Δ*tmk1* mutant colonies is due to enhanced cell wall stress provoked by the observed constitutive up-regulation of chitinase gene expression in the mutants. This would be consistent with previous studies showing that most *T. atroviride* chitinases are not only involved in the degradation of host cell walls during mycoparasitism but are also required for rebuilding and recycling of the fungus' own cell wall during growth [62]. Anyway, whereas in *N. crassa* the cell wall integrity MAPK has been shown to act downstream of the Mak-2/PP-1 pathway during cell fusion [45] this seems not to be the case in *T. atroviride*.

Comparable to hyphal anastomosis, which roughly consists of pre-contact sensing, chemotropism, adhesion, and subsequent cell wall lysis at the fusion site [63], the mycoparasitic fungus-fungus interaction comprises similar steps. The initial interaction between *Trichoderma* and the host fungus is characterized by chemotropic growth of the mycoparasites hyphae towards the host [64] followed by the induction of a set of genes already before direct contact between the two fungi [65], [66]. The pre-contact induction of certain genes being involved in host lysis such as the *ech42* endochitinase and the *prb1* protease ([3], [67] led to a model in which degradation products of the hosts' cell wall are sensed by respective *Trichoderma* receptors. The resulting activation of intracellular signaling cascades by diffusible host-derived signals and, upon direct contact, lectins on the host surface, finally leads to the full induction of the mycoparasitic response resulting in host attack and lysis [4], [5]. In contrast to basidiomycetous fusion parasites [68] and the mycoparasitic interaction between the two zygomycetes *Absidia glauca* and *Parasitella parasitica*, where fusion bridges between the two fungi are formed [69], *Trichoderma* mycoparasites invasively grow inside the host. Ultrastructural studies revealed that coiling hyphae of *Trichoderma* constrict and partially digest the host cell wall at the interaction site followed by penetration and growth of *Trichoderma* invading hyphae inside the host [64]. Nevertheless, our phenotypic characterization of Δ*tmk1* and Δ*ste12* mutants revealed that Tmk1 and Ste12 play important roles in hyphal anastomosis as well as mycoparasitism in *T. atroviride*. The finding that Δ*tmk1* and Δ*ste12* mutants not only showed enhanced attachment to own and foreign hyphae but also to hyphal-like plain nylon fibers suggests a repressing role of the Tmk1 signaling pathway on hyphal attachment in the absence of respective (host-derived) signals. Taking into consideration that cAMP stimulates coiling in *T. atroviride* [70] and that Δ*tga3* mutants missing the adenylate cyclase-stimulating Gα subunit Tga3 are defective in host recognition and coiling [71], the Tmk1 MAPK cascade and the cAMP pathway seem to have antagonistic roles in regulating the mycoparasitism-relevant coiling response.

In *M. oryzae*, the Mcm1 orthologue MoMcm1 was found to interact with the Mst12 transcription factor. The findings that a *Momcm1 mst12* double mutant formed appressoria even on hydrophilic surfaces whereas this was not the case in *Momcm1* and *mst12* single mutants suggested overlapping functions of MoMcm1 and Mst12 in suppressing appressorium formation under non-conducive conditions [72]. In contrast to *M. oryzae*, the Mcm1 othologue seems to be dispensable for mediating the repressive role of Tmk1 on attachment to and coiling around hyphae and hyphal-like structures in the absence of respective signals in *T. atroviride*.

Deletion of *tmk1* or *ste12* resulted in an aberrant, i.e. host-independent over-expression of the *ech42* and *nag1* chitinases and the *prb1* protease. It is interesting to note that, despite these enhancements, Δ*tmk1* and Δ*ste12* mutants are impaired in both hyphal fusion and mycoparasitic host lysis which suggests a co-regulation of these processes by this signaling pathway and the involvement of additional still uncharacterized genes in *T. atroviride* mycoparasitism. Otherwise, the reduced mycoparasitic abilities of the mutants may result from their failure to build a fully interconnected mycelial network as has previously been predicted to be the cause of the reduced pathogenicity displayed by MAPK mutants of plant pathogens [60].

Detailed analyses of the signals originating from host fungi and their respective *Trichoderma* receptors together with a functional analysis of the downstream targets of conserved signaling cascades will be necessary to fully understand the mycoparasitic fungus-fungus interaction. Comparative genome analysis revealed that transcription factors are expanded in the mycoparasites *T. atroviride* and *T. virens* compared to the saprophyte *T. reesei* [73]. However, there are only few reports on the functional characterization of genes encoding proteins with transcription factor activity from mycoparasitic *Trichoderma* species [74]–[77]. This study revealed an essential role of the Ste12 transcriptional regulator in mediating the output of the mycoparasitism-relevant Tmk1 MAP kinase pathway (Figure 9) and illustrated the interconnection between hyphal anastomosis and the mycoparasitic activity of *T. atroviride*.

## Supporting Information

Figure S1 **Genotypic analysis of Δ** ***ste12*** **gene deletion and complementation mutants.** (A) PCR analysis of the three out of 20 hygromycinB-resistant transformants that showed a stable integration of the *ste12* deletion construct after three rounds of single spore isolation. The primer pair hph-FW and hph-RV (Table 1) amplified a 560-bp fragment of the integrated *hph* gene. (B) Southern hydridization of *Nco*I-digested DNA from parental strain (WT) and the three different putative deletion mutants (D, F, S) with a 2693-bp probe covering 1415-bp of the 5′ non-coding region of the *ste12* gene and 1278-bp of the *hph* selection marker cassette. The parental strain and transformants F and S show a 1597-bp band indicative of the native *ste12* gene, while transformant D lacks this band and instead shows two bands of 2314-bp and 3360-bp confirming transformant D as a *ste12* null mutant resulting from homologous recombination at the *ste12* locus. (C) Confirmation of complementation mutants by PCR using primers ste12-C-FW and ste12-C-RV (Table 1) located 1500-bp 5′ and 3′, respectively, of the *ste12* open reading frame. This primer pair is expected to amplify a 5141-bp fragment in the parental strain (lane 5) and a 6159-bp fragment in the Δ*ste12* mutant (lanes 1 and 4). The amplification of both fragments in complementation mutant *ste12*-C1 (lane 2) confirms ectopic integration of *ste12*, whereas the presence of only the 5141-bp band in complementation mutant *ste12*-C2 (lane 3) is indicative of a rescue of the *ste12* gene at the homologous locus by replacement of the deletion construct. (D) RT-PCR with primers ste12-FW and ste12-RV (Table 1) amplified the expected 320-bp fragment of the *ste12* gene in the parental strain (lane 3), the Δ*tmk1* mutant (lane 2), and the *ste12* complemented strains (lanes 4 and 5) but not in the Δ*ste12* deletion mutant (lane 1).
(TIF)
