# Supplementary figures and images for: The Transcription Factor Ste12 Mediates the Regulatory Role of the Tmk1 MAP Kinase in Mycoparasitism and Vegetative Hyphal Fusion in the Filamentous Fungus Trichoderma atroviride
Source: PLoS One. 2014 Oct 30;9(10):e111636. doi: 10.1371/journal.pone.0111636 (PMC4214791; doi:10.1371/journal.pone.0111636)

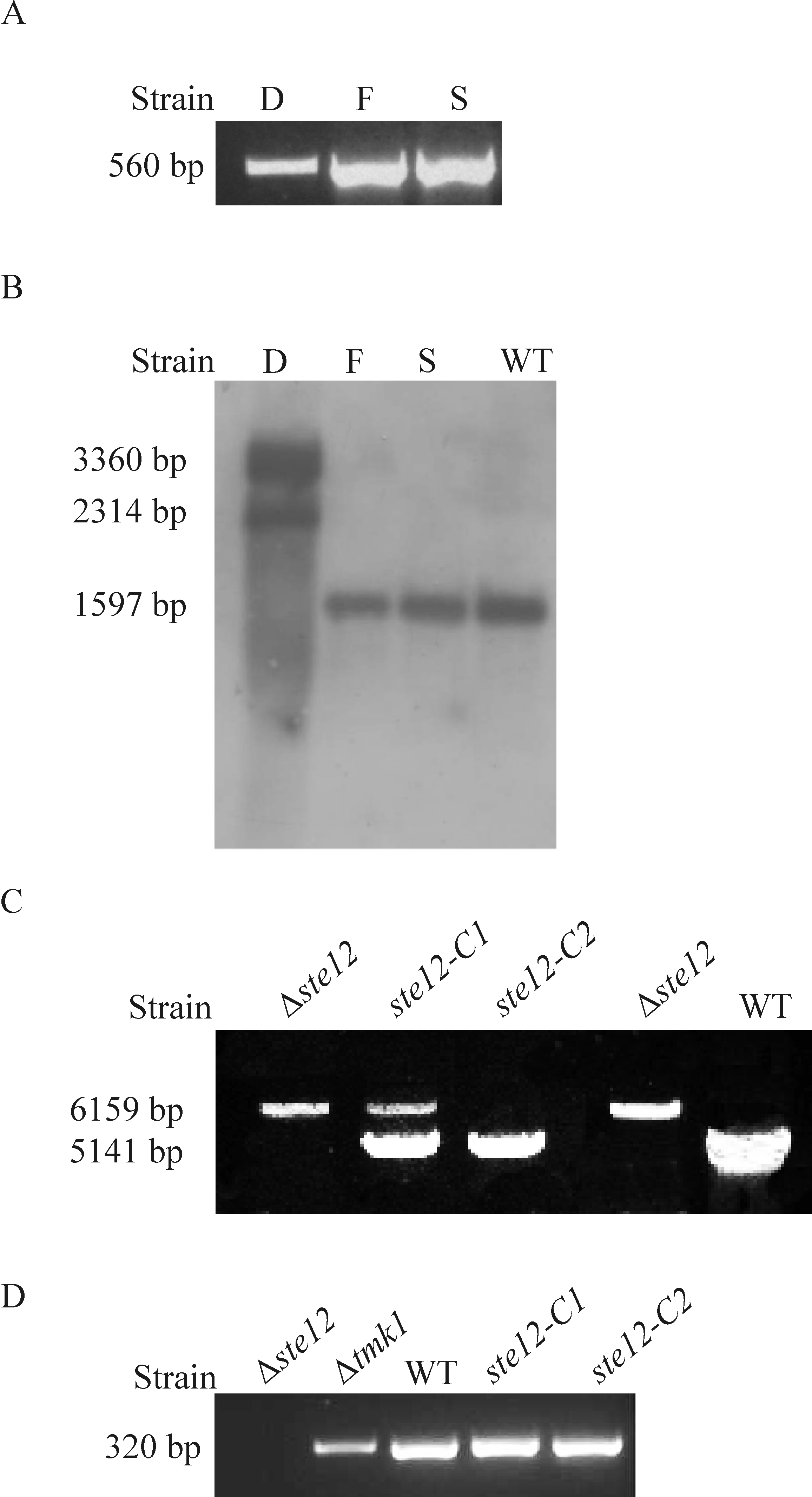

Supplement: Figure S1 — Genotypic analysis of Δ ste12 gene deletion and complementation mutants. (A) PCR analysis of the three out of 20 hygromycinB-resistant transformants that showed a stable integration of the ste12 deletion construct after three rounds of single spore isolation. The primer pair hph-FW and hph-RV (Table 1) amplified a 560-bp fragment of the integrated hph gene. (B) Southern hydridization of NcoI-digested DNA from parental strain (WT) and the three different putative deletion mutants (D, F, S) with a 2693-bp probe covering 1415-bp of the 5′ non-coding region of the ste12 gene and 1278-bp of the hph selection marker cassette. The parental strain and transformants F and S show a 1597-bp band indicative of the native ste12 gene, while transformant D lacks this band and instead shows two bands of 2314-bp and 3360-bp confirming transformant D as a ste12 null mutant resulting from homologous recombination at the ste12 locus. (C) Confirmation of complementation mutants by PCR using primers ste12-C-FW and ste12-C-RV (Table 1) located 1500-bp 5′ and 3′, respectively, of the ste12 open reading frame. This primer pair is expected to amplify a 5141-bp fragment in the parental strain (lane 5) and a 6159-bp fragment in the Δste12 mutant (lanes 1 and 4). The amplification of both fragments in complementation mutant ste12-C1 (lane 2) confirms ectopic integration of ste12, whereas the presence of only the 5141-bp band in complementation mutant ste12-C2 (lane 3) is indicative of a rescue of the ste12 gene at the homologous locus by replacement of the deletion construct. (D) RT-PCR with primers ste12-FW and ste12-RV (Table 1) amplified the expected 320-bp fragment of the ste12 gene in the parental strain (lane 3), the Δtmk1 mutant (lane 2), and the ste12 complemented strains (lanes 4 and 5) but not in the Δste12 deletion mutant (lane 1). (TIF) [file pone.0111636.s001.tif]
